# Comparison of prone vs. supine unenhanced CT imaging in patients with clinically suspected ureterolithiasis

**DOI:** 10.1007/s00261-016-0918-1

**Published:** 2016-09-26

**Authors:** Matthias Meissnitzer, Thomas Meissnitzer, Stephan Hruby, Stefan Hecht, Andreas Gutzeit, Laura Holzer-Frühwald, Klaus Hergan, Rosemarie Forstner

**Affiliations:** 1 Department of Radiology, Landeskrankenhaus Salzburg, Paracelsus Medical University, Müllner-Hauptstrasse 48, 5020 Salzburg, Austria; 2 Department of Urology, Landeskrankenhaus Salzburg, Paracelsus Medical University, Müllner-Hauptstrasse 48, 5020 Salzburg, Austria

**Keywords:** Computed tomography, Urolithiasis, Ureteral calculi, Supine position, Prone position

## Abstract

**Purpose:**

To retrospectively evaluate whether prone CT scanning is superior to supine scanning for correct localization of distal urinary calculi in patients with acute flank pain.

**Methods:**

Consecutively performed unenhanced CT scans in patients with acute flank pain were retrospectively analyzed in 150 patients in supine and another 150 patients in prone position. Images were reviewed by two radiologists on consensus. Findings in both groups were compared using two-sided Fisher Exact tests and Wilcoxon–Mann–Whitney test.

**Results:**

Urinary calculi were found in 67% of patients in each group. In the supine scanning group, there were 16 cases, in which the location of the stone was equivocal being either located intramurally at the ureterovesical junction (UVJ) or having already passed into the bladder. In contrast, in the prone imaging group all distal stones could be allocated accurately, either to the intramural UVJ or the urinary bladder (37 intramural UVJ stones and six bladder stones in prone scanning group vs. 21 intramural UVJ stones and one bladder stone when scanned supine).

**Conclusion:**

Prone scanning is superior to supine CT scanning for acute flank pain to accurately distinguish intramural UVJ stones from stones that have already passed into the bladder, a distinction which influences patient management.

Unenhanced multidetector computed tomography (CT) has become the diagnostic modality of choice for urinary stone detection in patients presenting with acute flank pain [1, 2]. It has been demonstrated that CT combines superb diagnostic capabilities with sensitivities and specificities approaching almost 100% with a wide availability [3, 4]. Furthermore, unenhanced CT can be easily performed in the acute setting and is highly reproducible [5]. In addition to symptoms and clinical factors, stone size and location—as visualized in CT—are central parameters to guide treatment decision [1, 2]. Patients can either be managed conservatively if stones are small and more distally located or endoscopically in case of larger or persistent stones [1, 2]. For stones located at the region of the ureterovesical junction (UVJ), it is important to determine whether the calculus is located intramurally at the UVJ or has already passed into the bladder (Fig. 1). In the latter, stones may simply lie posterior in the bladder adjacent to the ureteral ostium mimicking an intramural position. However, this distinction of the exact location of a distal stone is crucial for patient management, because prognosis and therapy are different [1, 2]. Therefore, a CT exam should provide an answer to the question whether the calculus is still located in the intramural UVJ or has already passed into the urinary bladder. It has been demonstrated that this distinction cannot be made clinically or by evaluating CT scans for secondary signs of obstruction such as dilatation of ureter and renal pelvis, periureteral and perirenal stranding, or edematous renal enlargement [6]. Levine et al. demonstrated that the location of a considerable number of distal calculi remains equivocal in conventional supine CT scans (Fig. 2). They suggested that additional prone scanning is needed in these cases to accurately locate stones to UVJ or urinary bladder [6]. Due to their work and upon expert consensus at our department, we changed the standard protocol for patients referred to CT imaging with acute flank pain from supine to prone scanning effective from June 2008. This retrospective review was designed to further validate prone scanning as standard protocol in 150 consecutive patients with acute flank pain and to perform a comparison to 150 consecutively performed supine scans for the same indication before changing the protocol.

**Fig. 1:**
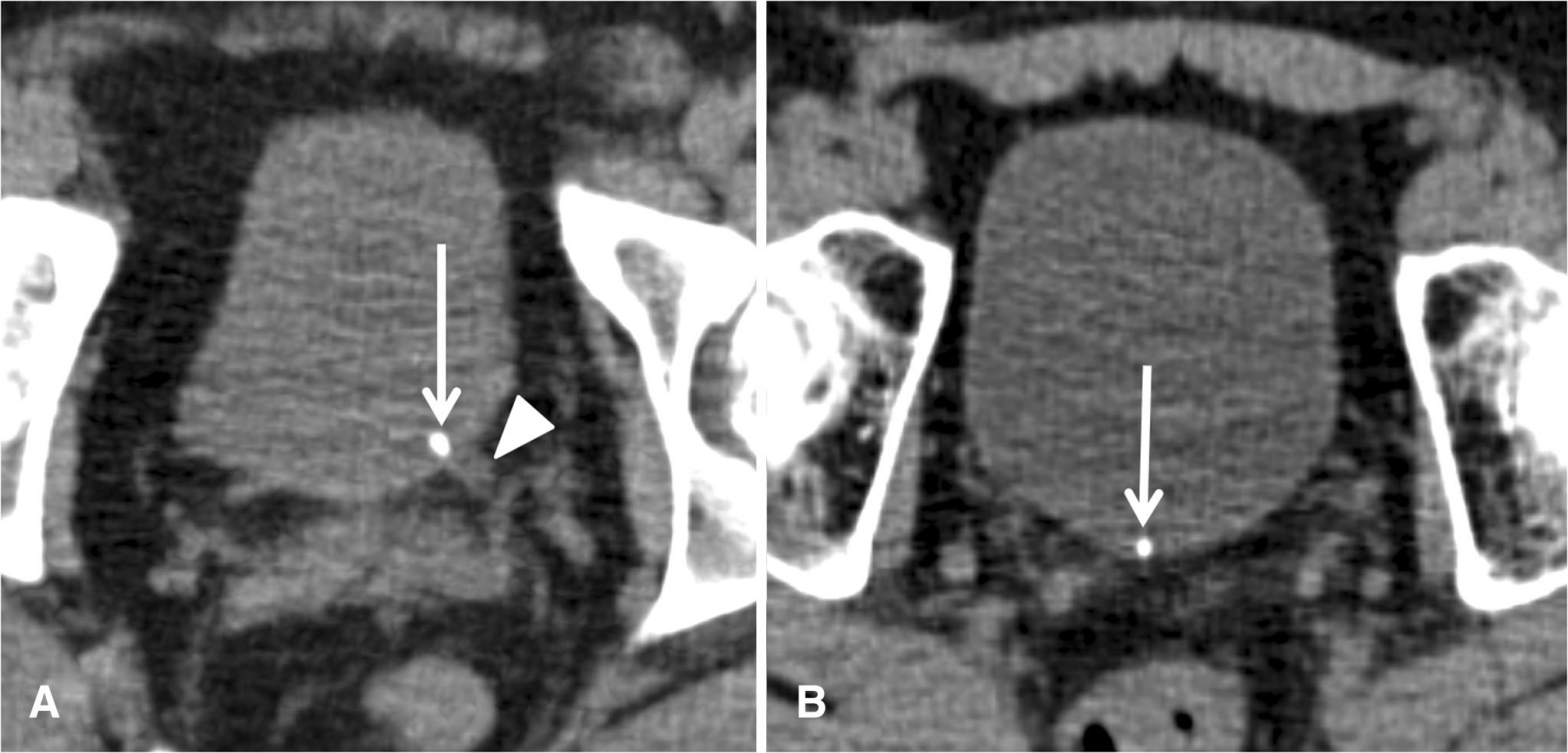
**A**, **B** Supine axial unenhanced CT images in two different patients with acute left-sided renal colic and a stone clearly impacted at the UVJ (*arrow* in **A**), dilated distal left ureter (*arrowhead* in **A**), and a ureteral stone that had unequivocally already passed into the urinary bladder (*arrow* in **B**) and is located posteriorly in the bladder more to the midline

**Fig. 2:**
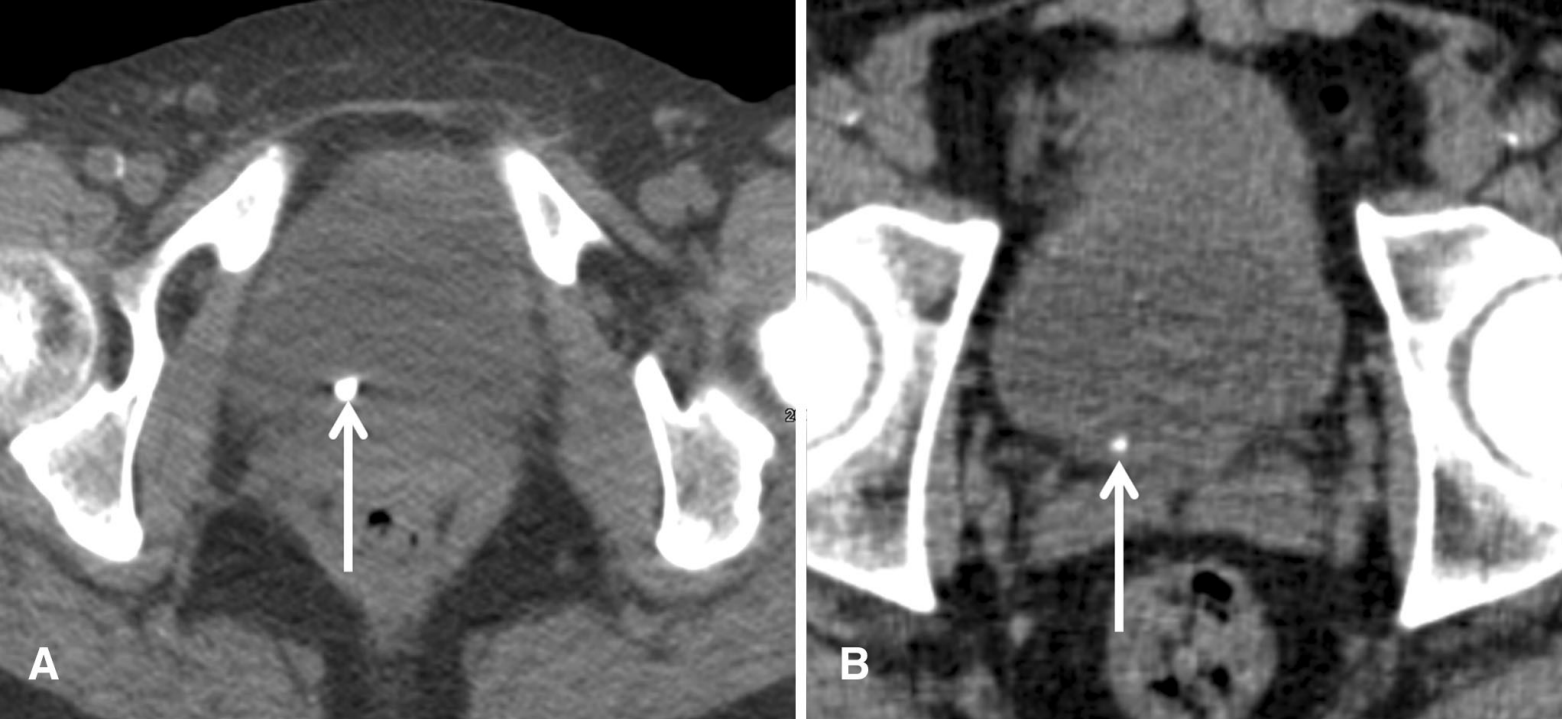
**A**, **B** Supine axial unenhanced CT images in two different patients with right-sided acute renal colic and stones in equivocal distal location on CT. On follow-up cystoscopy, the stone in (**A**) was located in the urinary bladder; the stone in (**B**) was extracted from the UVJ

## Patients and methods

At our institution, standard protocol for unenhanced CT imaging of patients with clinically suspected urolithiasis was changed from supine to prone scanning on June 1, 2008. This was performed on expert consensus among urologists and radiologists. We have entirely eliminated ureteral stone findings with equivocal distal anatomic location ever since. Retrospectively, 300 consecutively performed CT exams were retrieved for this study from the picture archiving and communication system (PACS). The study protocol was waived by the responsible local ethics committee. Retrospective inclusion identified 150 unenhanced CT exams performed with the patient lying supine between September 12, 2007 and June 1, 2008. The protocol was then changed, and 150 consecutive exams were identified and performed between June 2, 2008 and March 24, 2009 with the patient lying prone during the exam. A statistical comparison of the two groups was performed. Inclusion criteria were retrospectively identified patients presenting with renal colic, unilateral flank pain, and referral to unenhanced CT imaging to evaluate for urolithiasis. Clinical evaluation and sonography of the kidneys and urinary bladder had been performed before referral to CT imaging. CT exams were performed on one of two multidetector (MD) CT scanners (64-MDCT Philips Brilliance, Philips Healthcare, Best, The Netherlands, and 128-MDCT Siemens AS+, Siemens Healthcare, Erlangen, Germany). The choice on the type of scanner to perform the CT exam was at the discretion of the responsible technician. After obtaining a scout image, the scan range was set from the level of the diaphragm to the pubic symphysis. Images were obtained with a constant tube voltage of 120 kV and a tube amperage-time-product of 60 mAs according to a’low-dose’ protocol for an average patient. However, it was also at the discretion of the technician to adapt the mAs depending on the patient’s habitus. Reformations included 3-mm axial and coronal images. For image analysis, all CT images obtained in prone positioning were automatically flipped 180° and thus could be read in the usual way.

**Fig. 3:**
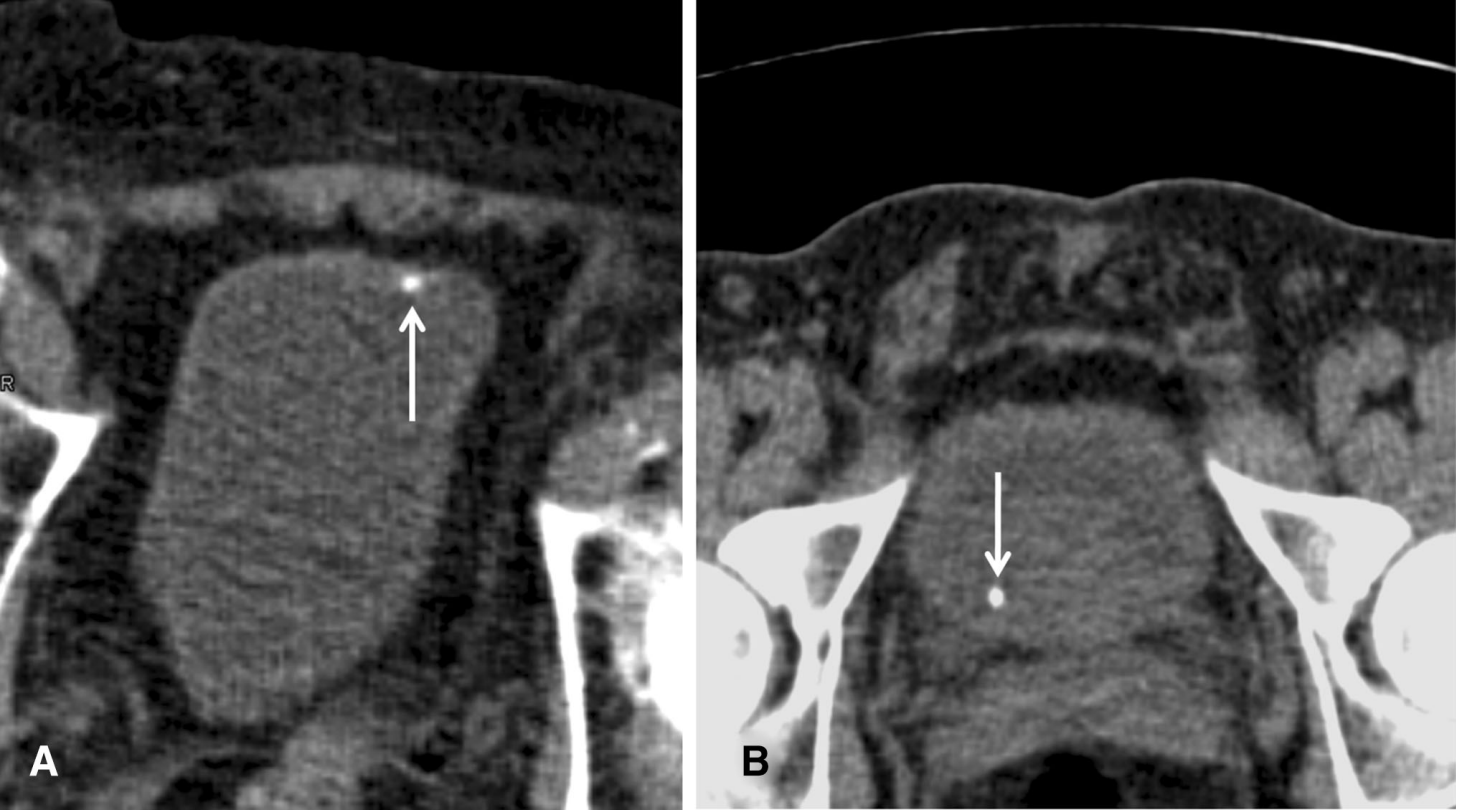
**A**, **B** Prone axial unenhanced CT images in two different patients with acute renal colic (images were flipped 180° and can be read in the usual way). Stone located anteriorly in the bladder in keeping with a ureteral stone that had already passed into the bladder (**A**, *arrow*). Intramural stone impacted at the ureterovesical junction not moving anteriorly on the prone scan (**B**, *arrow*)

Patient data of all identified exams were entered into an MS Excel data sheet. Images were reviewed for the purpose of this study in retrospect by two readers with regard to the presence or absence of urolithiasis and the location of the clinically most relevant stone causing the unilateral flank pain. The readers were a board-certified radiologist (MM) and a radiology resident (HS) with 5 and 2 years of experience in reading unenhanced CT exams for urolithiasis. All cases were reviewed by both readers, and images were discussed until a consensus for each case was reached. The readers were blinded to the original reports. Special consideration was given to the following criteria: the presence or absence of stones at the intramural UVJ, stones that had passed into the bladder, or stones that could not be unequivocally located to intramural UVJ vs. being located inside the bladder close to the ureteral ostium. Criteria for differentiating UVJ stones from stones that have already passed into the urinary bladder on supine CT scans included stone location clearly within the distal ureteral ostium or slightly protruding into the bladder lumen indicating UVJ stones. In supine scanning, if a stone appeared to be lying posteriorly and dependently in the urinary bladder without being clearly trapped at the UVJ or moving more to the midline, the location of the urinary calculus was classified as equivocal (Figs. 1, 2). In cases in which prone scanning was performed, the criteria for a urinary bladder stone were anterior location within the bladder suggesting mobility of the calculus vs. being trapped at the UVJ when located posteriorly (Fig. 3). In 7 patients of both groups with a calculus unequivocally located in the bladder, the presence or absence of secondary signs of obstruction on the symptomatic side such as dilatation of the renal pelvis, perinephric and periureteric stranding, and edematous renal enlargement were noted. For all cases with intramural UVJ stones, equivocal stones, and urinary bladder stones, stone size was measured on CT, and follow-up data, with regard to whether or not cystoscopy for stone removal was performed, were retrieved from the medical records.

Statistical analysis was performed using two-sided Fisher Exact tests, for comparison of the prone and supine scanning groups. Wilcoxon–Mann–Whitney test for singly ordered cross tabulations was used to test the significance of localization vs. treatment (conservative/cystoscopy for stone removal). Significance levels were set at 0.05 (5%).

## Results

Results of prone and supine scanning groups are summarized in Table 1. In both groups, the male–female ratio was similar to 90/60 in the prone imaging group and 87/63 in the supine imaging group (*p* = 0.81). Average age was 50 years in the prone imaging group and 49 years in the supine imaging group. Unenhanced CT imaging excluded urolithiasis in exactly the same number of patients in both groups (50 patients in the supine vs. 50 patients in the prone group). In the remaining 100 exams in each group, CT identified at least one urinary calculus. Among the 300 cases, the likelihood of diagnosing urinary calculi therefore was 66.67% (200/300 cases); it was higher in male vs. female patients with 76.8% vs. 52.5% (136/177 vs. 64/123, *p* < 0.001). In the conventional supine imaging group, there were 21 cases (21%) with stones unequivocally located at the UVJ and 1 patient with a stone that had already passed into the bladder. However, there were 16 cases (16%) where the exact anatomic location of the urinary calculus on the symptomatic side was equivocal, being either at the intramural UVJ or close to the ureteral ostium inside the urinary bladder. In the prone imaging group, all stones could be unequivocally located to the intramural UVJ (37 cases, 37%) or to the urinary bladder (6 cases, 6%), and so there were no cases with equivocal stone location with regard to UVJ vs. urinary bladder (Table 1). Within the period in which the 150 prone CT exams were retrospectively included, there was only one patient on whom prone imaging was not possible. This patient did not tolerate lying prone during the exam due to severe skeletal deformity from ankylosing spondylitis, and thus was not included in the study. Therefore, the vast majority of patients of 99.3% (150 of 151) tolerated prone scanning.

**Table 1:** Comparison of the supine and prone scanning groups

	Supine imaging	Prone imaging	*p* value
Number of patients	150	150	
Male/female	87/63	90/60	0.81
Average age	49.62 years	50.02 years	
Patients without stones	50	50	1.0
Patients with at least 1 stone	100	100	1.0
Unequivocal intramural UVJ stone	21	37	0.019
Stone in equivocal location (intramural UVJ vs. urinary bladder)	16	0	<0.0001
Unequivocal urinary bladder stone (ventral or dorsal)	1	6	0.12
Stone located proximally to UVJ	62	57	0.39

Follow-up data for 58 UVJ stones, 16 equivocal UVJ stones, and seven urinary bladder stones are provided in Table 2. Among the three groups, cystoscopy for stone removal was performed in 36%, 25%, and 0% (*p* = 0.071). Among 16 patients in whom the location of a urinary calculus was equivocal (intramural UVJ vs. location in bladder) from the unenhanced supine CT scan, there was conservative treatment in 12 without any further imaging or intervention (Table 3). The remaining four patients underwent cystoscopy. Among three of them, a calculus was extracted from the intramural UVJ, and in the remaining patient, a calculus was found in the urinary bladder, having also either been located inside the urinary bladder at the time of the supine CT scan or having passed into the bladder between CT imaging and cystoscopy.

**Table 2:** Management of urinary stones according to their presumed location, conservative vs. cystoscopy for stone removal

	Conservative	Cystoscopy	*p*-value
UVJ stones (*N* = 58)	37 (64%)	21 (36%)	0.071
Equivocal UVJ stones (*N* = 16)	12 (75%)	4 (25%)	
Urinary bladder stones (*N* = 7)	7 (100%)	0 (0%)

**Table 3:** Follow-up of 16 patients with stones in whom the stone location was equivocal in supine CT imaging

Number of patients	Follow-up
12	Conservative treatment without further imaging, finally complete remission of symptoms
3	Cystoscopy with stone removal from intramural UVJ
1	Cystoscopy with stone located in urinary bladder, no stone at intramural UVJ

Stone size as measured on CT for UVJ, equivocal UVJ, and urinary bladder stones is given in Table 4. There were no statistically significant differences in size (*p* = 0.26) between UVJ stones (4.22 ± 1.52 mm), equivocal UVJ stones (4 ± 1.54 mm), and urinary bladder stones (3.29 ± 0.76 mm).

**Table 4:** Stone size for UVJ, equivocal UVJ, and urinary bladder stones

	Stone size (Mean ± STD in mm)	*p*-value
UVJ stones (*N* = 58)	4.22 ± 1.52	0.260
Equivocal UVJ stones (*N* = 16)	4 ± 1.54	
Urinary bladder stones (*N* = 7)	3.29 ± 0.76	

The assumed anatomic locations of the urinary stones in the supine and prone imaging group were statistically compared with two-sided Fisher Exact tests; the significance level was set at 0.05. Prone scanning eliminated equivocally located stone findings completely (*p* < 0.0001), and more stones could be allocated to the intramural UVJ (*p* = 0.019) and the urinary bladder (*p* = 0.12). The latter was not statistically significant; however, there was a trend that more stones could be allocated to the urinary bladder with prone scanning. Therefore, there was a statistically significant difference between the ability to locate stones with regard to the UVJ regions in the two groups.

Among 150 prone scans, 86 (57%) were performed on a 64-slice Philips scanner and 64 (43%) on the 128-slice Siemens scanner; however, among 150 supine scans, 50 (33%) were performed on the Philips scanner and 100 (67%) on the Siemens scanner. Therefore, there was a statistical significant difference (*p* < 0.001) with more scans being performed on the Philips scanner after changing the protocol. The decision on the type of scanner to perform the exam was at the discretion of the responsible technician. Due to organizational changes in the technicians’ workflow during the period for which we included patients, more patients were scanned on the Philips machine after changing the protocol.

Evaluation for the presence or absence of secondary signs of obstruction on the symptomatic side in seven patients, in whom the stone was unequivocally located in the bladder (among them six cases diagnosed with the patient lying prone and one case diagnosed with the patient lying supine during the CT exam), revealed secondary signs in five cases (71%).

## Discussion

Urolithiasis has become a common entity, reaching a prevalence of up to 20%, particularly in developed countries [1, 7]. For example, an estimated 1.2 million Americans and 0.75 million Germans are being affected annually [8, 9]. Unenhanced CT has become the standard diagnostic test and serves as the gold standard for rapid diagnosis of acute flank pain using low-dose protocols to reduce the associated radiation risk [5]. A meta-analysis of low-dose CT for suspected urolithiasis showed pooled sensitivity of 97% and specificity of 95%, and underlines the superb performance of CT [3, 4]. In addition to the excellent diagnostic accuracy, unenhanced CT is widely available and can easily be performed without requiring patient preparation and laboratory data [1, 2].

More recently, there has been concern about the increasing use of unenhanced CT for the detection of urolithiasis with little yield in certain subgroups, especially young women [10]. The overall likelihood of stone detection in our cohort is high with 66.67%; it is lower in females than in males (52.5% vs. 76.8%, *p* < 0.001). However, the positive stone rate in females is higher than reported in the literature, which could be attributed to the performance of a thorough ultrasound exam in female patients that may have obviated the need for CT imaging in these patients [10].

Stone size and location are important management parameters in acute renal colic [1, 2]. Stone size as defined by the maximal diameter and stone location can be revealed by unenhanced CT and are main determinants for patient treatment. Smaller and more distally located ureteral stones are more likely to pass spontaneously. About 95% of stones smaller than 5 mm were reported to pass spontaneously within a few weeks, and therefore are mostly treated conservatively [11]. Urinary calculi above 6 mm are usually managed actively; however, there is no exact cut-off size when active treatment should be performed. The current European Association of Urology Guidelines suggest conservative management for stones smaller than 6 mm in size based on expert consensus due to a significantly higher stone expulsion rate and shorter expulsion time for stones below 6 mm [1, 12–14].

Stone impaction most often occurs at sites of extrinsic obstruction, physiological luminal narrowing, or at locations with a change in ureteral course [13, 14]. According to the literature, up to 18% of patients with acute flank pain and obstructing ureteral stones present with stones at the UVJ [6]. In the presented prone imaging cohort, we found stones unequivocally located at the UVJ in an even higher rate of 37% (Table 1). If a stone is located in the region of the UVJ, it is crucial to differentiate intramural stones at the ureteral orifice from stones that have already passed into the urinary bladder, and thus simply lie posterior in the bladder close to the ureteral orifice. Intramural stones located at the UVJ might require treatment, whereas stones that have already passed into the bladder generally require no treatment. These patients are only instructed to strain their urine in order to retrieve the calculus for chemical analysis [1, 2, 6].

It has been shown that supine CT imaging cannot reliably distinguish stones impacted at the UVJ from stones that have already passed into the bladder (Fig. 2) [6]. The study by Levine et al. also revealed that secondary signs of obstruction such as dilatation of the renal pelvis, perinephric and periureteric stranding, and edematous renal enlargement are not useful for this distinction because these signs may persist even after stone passage in up to 50% of patients [6]. This was also found in our study cohort with the presence of secondary signs in 71% (5/7) of patients with ureteral stones that had unequivocally already passed into the bladder. Thus, in ureteral calculi that had unequivocally already passed into the bladder, secondary signs were present in the majority of cases. Furthermore, clinical signs and symptoms cannot be used to differentiate obstructed UVJ stones from bladder stones confidently, since pain may persist despite stone passage [6]. On the other hand, smaller stones obstructed at the UVJ may only cause mild or intermittent symptoms, despite being obstructive.

Among 150 supine CT exams in the acute setting, there were 16 CT studies with stones in an equivocal location being either impacted at the UVJ or having passed into the urinary bladder. It has been demonstrated that additional prone or lateral decubitus imaging can be used to make this distinction showing the mobility of calculi located in the urinary bladder [6]. If a stone moves to the dependant anterior aspect of the bladder, this finding indicates that it has already passed into the urinary bladder (Fig. 3). However, the need for additional imaging increases patient dose and requires the immediate supervision of the supine scan by a radiologist to decide whether additional images need to be obtained. If the radiologist is not able to review a supine CT scan immediately, the patient might already have left the Radiology department, and imaging findings might be inconclusive or the report may be inaccurate, resulting in a delay in diagnosis. Furthermore, we assume that additional scanning increases radiation dose and may lead to patient and staff inconvenience, requiring an additional logistic effort and presumably also increasing costs.

Our retrospective comparison of 150 prone and 150 supine consecutively performed CT scans for acute flank pain clearly shows that prone imaging eliminates stone findings with equivocal anatomic location in the region of the UVJ (Table 1).

The clinical relevance of accurately locating distal urinary calculi is reflected in the percentages of cases in which cystoscopy for stone removal was performed in our cohort (Table 2). While cystoscopy was performed for 21 (36%), among 58 cases in which the stone was unequivocally located to the UVJ on CT, no intervention was performed among seven ureteral calculi that had already and unequivocally passed into the urinary bladder at the time of CT imaging. Among 16 cases with equivocal stones, 4 (25%) underwent cystoscopy.

With the management trend shifting to primary endoscopic removal of ureteric calculi, we think that it is particularly important to accurately locate distal ureteral calculi [15–18].

Common adverse effects and implications of endoscopic stone removal include urinary tract infection, iatrogenic trauma, gross hematuria, or the necessity for stent placement and removal [16, 19]. Identifying those ureteral calculi that have already passed into the bladder may help avoid unnecessary intervention and related adverse effects or disadvantages.

Regarding the CT protocol, there was only one patient who did not tolerate lying prone after changing the standard protocol to prone scanning during the time period in which we retrospectively identified 150 prone CT exams. This patient suffered from severe vertebral deformity due to ankylosing spondylitis and was not included in the study. However, in the vast majority of 99.3% (150 of 151) of patients, performance of a prone scan was possible.

Levine et al. demonstrated in their study the impossibility to accurately distinguish obstructed UVJ stones from ureteral stones that had already passed into the urinary bladder, and they highlighted the importance of additional scanning for this differentiation [6]. Our approach differs to theirs in that we suggest prone CT scanning in patients with acute flank pain from the outset. Additionally, we demonstrate that lying prone during the CT scan is tolerated by the majority of patients.

We identified several limitations in this study. First, prone scanning and supine scanning were not performed in the same patients, and the study relies on statistical differences. Second, readers were not blinded to patient positioning (prone vs. supine scanning) as this can easily be derived from the images themselves (e.g., from bowel gas or fluid). Third, there was a statistically significant difference between prone and supine scanning in the use of the two CT scanners, since there was no standard protocol on which scanner to use. However, this difference was due to a change of the technicians’ workflow which occurred during the study period and may have introduced an additional bias. But on the radiological assessment of the exams, this difference had no effect.

To date, the current guidelines for CT imaging of urolithiases do not elaborate on whether or when prone imaging may be advantageous [1]. The clinical practice among institutions varies. Our work underlines the advantages of prone unenhanced CT scanning in all patients with acute flank pain and should influence future guidelines.

## Conclusion

Differentiation of intramural stones at the UVJ from those that have already passed into the urinary bladder may be impossible in conventional supine CT scans. However, besides clinical signs and symptoms and stone size, the location of a stone is a major determinant for patient management in acute urolithiasis. Routinely performing CT scans with the patient in prone position allows allocating distal urinary calculi easily and accurately. Prone CT scanning is tolerated by the majority of patients; it eliminates stone findings with equivocal anatomic location and obviates the need for additional imaging. Thus, our data suggest that with regard to accurately locating distal ureteral stones, prone CT scanning is superior to supine scanning.
